# Diversity and bioprospecting activities of endophytic Fungi associated with different Egyptian medicinal plants

**DOI:** 10.1038/s41598-025-01202-z

**Published:** 2025-06-03

**Authors:** Mohamed E. Osman, Alaa M. Abou-zeid, Mohamed A. Abu-Saied, Maha M. Ayid, Nessma A. El-Zawawy

**Affiliations:** 1 Department of Botany and Microbiology, Faculty of Science, Helwan University, Cairo, Egypt; 2 Department of Botany and Microbiology, Faculty of Science, Tanta University, Tanta, Egypt; 3 Polymeric Materials Research Department, Advanced Technology and New Materials Research Institute (ATNMRI), City of Scientific Research and Technological Applications (SRTA-City), New Borg El Arab, Alexandria Egypt; 4 Central Health Laboratories, Ministry of Health, Cairo, Egypt

**Keywords:** Medicinal plants, Fungal endophytes, Biodiversity, Antimicrobial, Cytotoxicity, Microbiology, Plant sciences

## Abstract

The use of medicinal plants in marginal communities was for the treatment of various ailments for centuries. Nevertheless, the potential of endophytic fungi (EF) associated with bioprospecting medicinal plants remains understudied. Research on the diversity of EF associated with various Egyptian medicinal plants remains limited. Therefore, our study conducted an analysis and comparison of the colonization frequency (CF), richness, and diversity indices of EF communities that inhabit nine different medicinal plants located in two different areas: the Protected Area of Wadi Degla in Maadi and the Natural Cultivated Area in Helwan, Egypt. These plants were *Agathophora alopecuroides*, *Anabasis setifera*, *Atriplex halimus*, *Halocnemum strobitaceum*, *Lantana camara*, *Mesembryanthemum forsskaollii*, *Raphanus raphanistrum*, *Suaeda vermiculata*, and *Zygophyllum coccineum*. Also, the antimicrobial and antioxidant potential of isolated EF has been investigated. A total of 39 morphospecies EF were isolated and identified, belonging to fifteen genera. *Aspergillus* spp. and *Penicillium* spp. were the dominant genera identified in the selected plants. *A. setifera* and *S. vermiculata* plants had the highest numbers of EF isolates, followed by *M. forsskaollii* and *R. raphanistrum*. Furthermore, these plants had a significant diversity index and species richness compared to other plants investigated. The most predominant EF was *Aspergillus* sp.3, which had the highest occurrence rate. Among all EF ethyl acetate extracts (EAEs), *Aspergillus* sp.3 demonstrated the highest antimicrobial activities against different human pathogenic bacteria, yeasts, and fungi. Furthermore, it showed the highest 2,2-diphenyl-1-picrylhydrazyl (DPPH) free radical scavenging activity. Therefore, this isolate was reidentified molecularly as *Aspergillus terreus* AUMC16223 with accession number PP491988. Moreover, EAE of *A. terreus* endophyte showed cytotoxicity potential activity with the significant IC_50_ value of 41.75 ± 1.83 µg/mL for the human lung carcinoma cell line (A549) and a nontoxic effect on the normal cell line (WI 38) with the significant CC_50_ value of 196.2 ± 3.74 µg/mL. Our results indicated diverse EF communities associated with different Egyptian medicinal plants, showing *A. terreus* endophyte extract as the most significant antimicrobial, antioxidant, and cytotoxic agent.

## Background

 Medicinal plants are the plants that are used to cure and prevent illnesses. Medicinal plants, in whole or in part, have a broad variety of therapeutic and commercial applications^1,2^. In addition, the pharmaceutical industry uses them as raw materials for natural pharmaceuticals in traditional medicine, particularly in traditional Indian and Chinese medicine^3^. These natural medications are vital for the healthcare needs of the developing world’s population^4–6^. Despite the rapid advancement of contemporary medicine, a significant number of therapeutic medications continue to be derived from natural compounds obtained from medicinal plants^3^. The clinical treatment employs different types of medicinal plants. Although wild medicinal plant resources are infrequent and highly desired, they are insufficient to meet market demand due to less reproductive capacity, overexploitation, and environmental stress^7,8^. Therefore, it has become essential to address the problem of enhancing the resources of medicinal plant germplasm. Endophytes are microbes that often live within plants and don’t manifest any symptoms of illness^9^. Recent studies have highlighted the significant role of endophytes in influencing the production and quality of medicinal plants through unique interactions between microbes and plants^10,11^. As a result of these symbiotic interactions, The plant modifies the metabolic processes of these endophytes to some degree, producing chemicals that may have defensive effects on both the host and the microbe^12^. In addition, endophytes directly or indirectly enhance the host plants’ growth and improve their stress resistance^13–15^.

Endophytic fungi (EF) have been receiving rapid attention because of their significant capacity to create a wide range of medicinally valuable metabolites that are similar to or better than those that originated from their host plant^12,16,17^. EF provides an abundant reservoir of new compounds, and those derived from medicinal plants thriving in certain ecological niches may possess a wide range of secondary metabolites with several biological activities such as antimicrobial, antioxidants, anticancer, antidiabetic, anti-inflammatory antiviral, immunosuppressive compounds, and insecticides and their use in the biosynthesis of nanoparticles^15,18–21^. Thus, studying endophytic fungi that live in medicinal plant species would provide plenty of chances to find novel metabolites with bioactive potential, to use in modern medicine, agriculture, and industry applications^22–25^.

The protected Area of Wadi Degla, a geologically ancient and steep-walled limestone canyon in Egypt’s Eastern Desert, is divided into three distinct physiographic ecological zones: upstream, midstream, and downstream^26^. The availability of water for plants is influenced by soil type, which varies along a wadi. Downstream and midstream regions of a wadi contain a blend of sandy and rocky soils, whereas the upstream area is characterized by predominantly sandy soil^27^.

The diversity, and distribution of the FE community are influenced by different factors such as soil type, climate, environmental conditions, tissue, genotype, age, location, and species of plant host^13,19,28,29^. The diversity of co-microbiota associated with medicinal plants is incomprehensible^30^. The special relationship between endophytes and medicinal plants should be better understood to enhance the quality of medicinal plants in Egypt. Consequently, in our study, we investigate the EF communities of different Egyptian medicinal plants and screen their biopotential activities.

## Results and discussion

### Isolation of endophytic fungi (EF)

Thirty-nine EF isolates were isolated from nine different healthy cultivated and wild medicinal plants located in two areas: the Natural Cultivated Area in Helwan (NCAH) and the Protected Area of Wadi Degla in Maadi, Egypt (PAWD). Fifteen morpho-genera were isolated, as shown in Table 1.

**Table 1:** Endophytic fungi (EF) isolated from different medicinal plants in different areas.

*N*	Plant location	Host plant, Voucher number	EF isolated	Species number	CF%
1	PAWD	*Agathophora alopecuroides* (010601)	*Aspergillus* sp. 1 *Chaetomium* sp. *Eurotium* sp. *Trichosporon* sp.	4	27.8
2	PAWD	*Anabasis setifera* (010600)	*Aspergillus* sp.2 *Aspergillus* sp.3 *Aspergillus* sp.4 *Fusarium* sp.1 *Penicillium* sp.1 *Penicillium* sp.2 *Phragmocamarosporium* sp. (1) *Neocosmospora* sp.	8	47.5
3	PAWD	*Atriplex halimus* L (005456)	*--*	*--*	*--*
4	PAWD	*Halocnemum strobitaceum* (005100)	*Aspergillus* sp.5 *Aspergillus* sp.6 *Phaeocremonium* sp. *Talaromyces* sp.	4	11.2
5	NCAH	*Lantana camara* (012505)	*Eurotium* sp. *Penicillium* sp.3 *Penicillium* sp.4	3	11.2
6	PAWD	*Mesembryanthemum forsskaollii* (010170)	*Aspergillus* sp.7 *Aspergillus* sp.3 *Curvularia* sp.1 *Phragmocamarosporium* sp. 2 Dark sterile mycelium sp.	5	33.5
7	NCAH	*Raphanus raphanistrum* subsp. *sativus* (013001)	*Aspergillus* sp.8. *Aspergillus* sp.3 *Curvularia* sp.2 *Fusarium* sp.2 *Penicillium* sp.5 *Rhodotorula* sp.	6	28
8	PAWD	*Suaeda vermiculata* (005144)	*Curvularia* sp.2 *Diaporthe* sp. *Penicillium* sp.6 *Ulocladium* sp.1 *Ulocladium* sp.2 Dark sterile mycelium sp.2	6	25
9	PAWD	*Zygophyllum coccineum* (000584)	*Aspergillus* sp.7 *Aspergillus* sp.3 *Quambalaria* sp.	3	8.4

PAWD* Protected Area of Wadi Degla, NCAH* Natural Cultivated Area in Helwan, CF%* Colonization frequency percent.

### Morphological identification and occurrence of EF

**Fig. 1:**
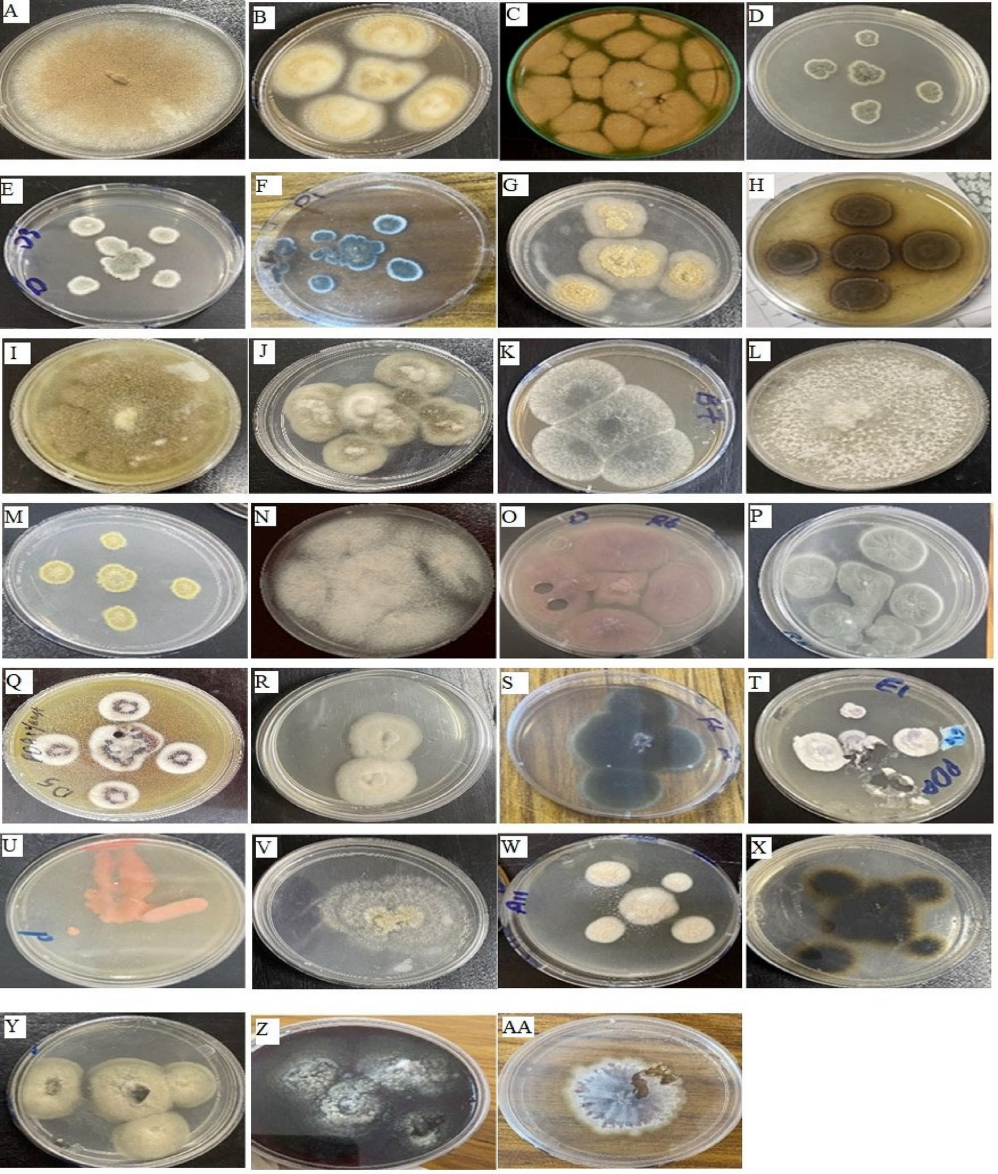
Pure cultures of some EF isolated from selected medicinal plants cultured on PDA plates for one week at 28 °C (**A**) *Aspergillus* sp.1, (**B**) *Aspergillus* sp.2, (**C**) *Aspergillus* sp.3, (**D**) *Aspergillus* sp.4, (**E**) *Aspergillus* sp.5, (**F**) *Aspergillus* sp.6, (**G**) *Aspergillus* sp.7, (**H**) *Aspergillus* sp.8, (**I**) *Chaetomium* sp., (**J**) *Curvularia* sp.1, (**K**) *Curvularia* sp.2, (**L**) *Diaporthe* sp., (**M**) *Eurotium* sp., (**N**) *Fusarium* sp.1, (**O**) *Fusarium* sp.2, (**P**) *Pencillium* sp.5, (**Q**) *Phaeocremonium* sp., (**R**) *Phragmocamarosporium* sp. 1, (**S**) *Phragmocamarosporium* sp. 2, (**T**) *Quambalaria* sp., (**U**) *Rhodotorula* sp., (**V**) *Talaromyces* sp., (**W**) *Trichosporon* sp., (**X**) *Ulocladium* sp.1, (**Y**)*Ulocladium* sp.2, (**Z**) Dark sterile mycelium sp. 1 and (**AA**) Dark sterile mycelium sp. 2.

**Fig. 2:**
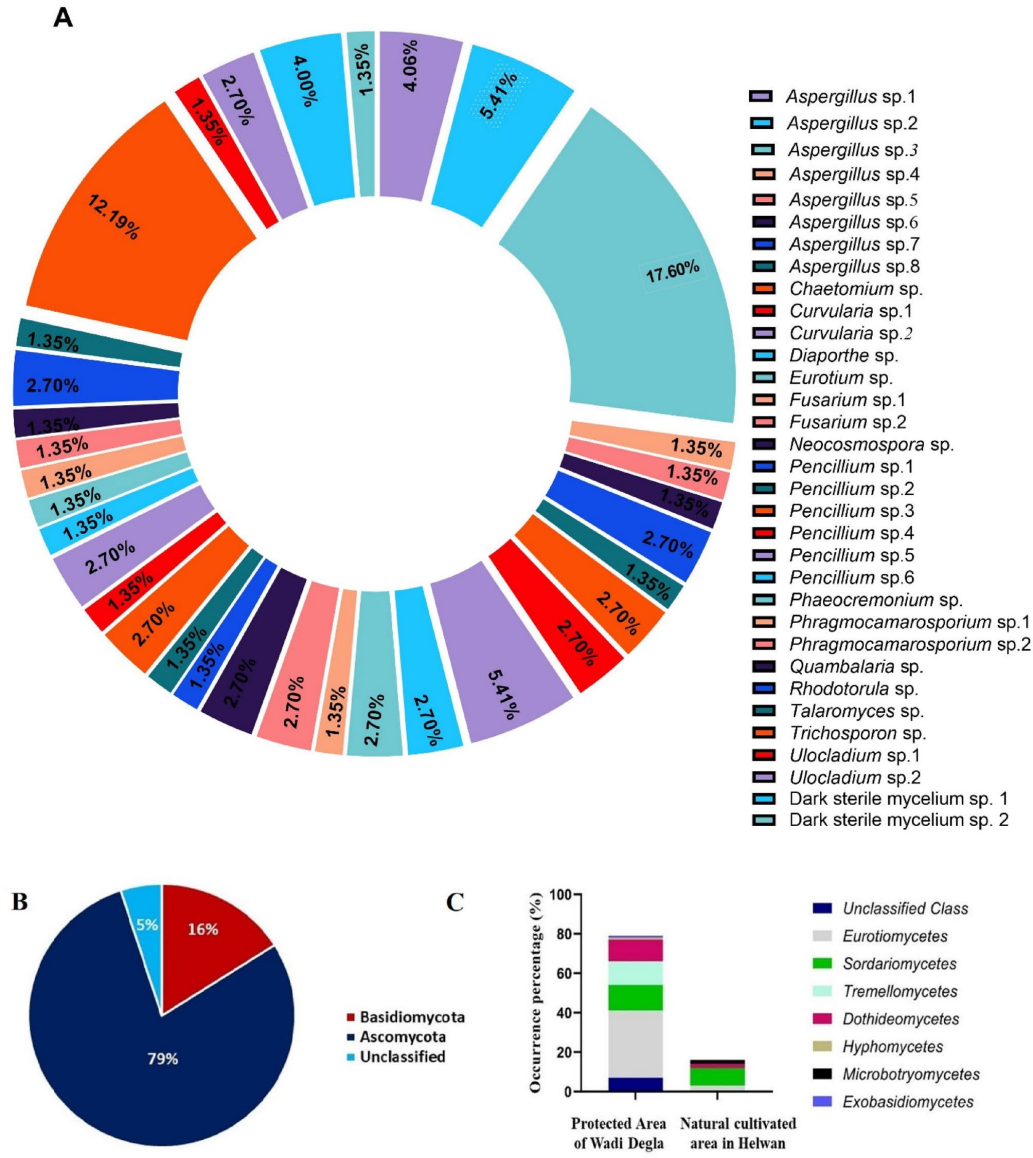
Taxonomical distribution of isolated EF, (**A**) Occurrence distribution of isolated EF species, (**B**) Occurrence distribution of EF at the phylum level, (**C**) Occurrence percent of various classes of isolated EF from different selected areas.

The species identification of 39 morphospecies EF was established morphologically, belonging to fifteen genera as shown in Fig. 1; Table 1. *Anabasis setifera* and *Suaeda vermiculata* plants had the highest numbers of EF isolates, as shown in Table 2. While no growth of EF appeared in the *Atriplex halimus* plant. Also, no growth of any microorganism appeared in the control sample, so the surface sterilization method was considered efficient. The most predominant genera identified were *Aspergillus* and *Penicillium*. Particularly, 12 EF isolates from *Aspergillus* spp. (34.64% occurrence), followed by *Penicillium* spp. with 6 isolates (10.64%). The other EF genera (54.72%) included *Chaetomium*, *Curvularia*,* Diaporthe*, *Eurotium*, *Fusarium*, *Neocosmospora*, *Phaeocremonium*, *Phragmocamarosporium*, *Quambalaria*, *Rhodotorula*,* Talaromyces*,* Trichosporon*, *Ulocladium*, and unclassified dark sterile mycelium (Fig. 2 A). Our results concurred with Osman et al.^31^ who revealed that the most prevalent EF isolates were the *Aspergillus* genus, followed by *Penicillium*. The isolated endophytic *Aspergillus* sp. 3 showed the highest incidence rate of 17.6%, as shown in Fig. 2 A. The isolated EF belonged to the Ascomycota phyla, showing the highest occurrence at 79%, followed by Basidiomycota at 16% (Fig. 2B). These results coincided with the earlier studies that the majority of isolated EFs were classified as Ascomycota^31,32^. Furthermore, Basidiomycota endophytes are rarely isolated^33^. As a result of comparing varieties of selected plants from different areas, 9 EFs (4 classes) were isolated from NCAH, while that of PAWD was 30 EFs (6 classes & 2 unclassified), as highlighted in Fig. 2 C.

Although there are no studies on EF associated with selected plants from a selected area in Egypt, there are a few studies on them in other countries. Our results coincided with Singh et al.^34^ who isolated *Alternaria* sp. endophyte from *Raphanus sativus* leaves. Also, Desire et al.^35^ isolated five EFs from *Lantana camara* leaves to biocontrol taro leaf disease. Moreover, Eldeghidy et al.^36^ isolated *A. terreus* and *A. flavus* endophytes from *Lantana camara* for camptothecin production with potential broad-spectrum anticancer activity. Moreover, Aletaha et al.^37^ isolated 5 EFs, *Aspergillus awamori*,* Aspergillus calidoustus*,* Rhizoctonia* sp., *Fusarium solani*, and *Fusarium* sp. from Iranian *Anabasis setifera*. Additionally, Jannati et al.^38^ isolated salt-resistant endophytic *Cladosporium limoniforme* from *Anabasis setifera*. Furthermore, BiBi et al.^39^ isolated rhizobacteria from *A. setifera* and *S. vermiculata* to improve the growth of plants under various conditions. Also, Kondrasheva et al.^40^ studied halotolerant endophytic *Penicillium* spp. from salt-tolerant *H. strobilaceum* to produce extracellularly various amounts of IAA under salt stress. Moreover, Alikulov et al.^41^ isolated endophytic bacteria from *H. strobilaceum* for the growth-promoting activity of cotton. As a result of rhizospheric arid soil studies, Boukelloul et al.^42^ isolated endophytic actinomycetes from halophytic *A. halimus* for inhibition of certain phytopathogenic fungi in vitro antagonism.

**Table 2:** Colonization frequency rate (CF%) of EF from different tissues of selected plants.

Host plants	Flowers %	Leaves %	Stems%	Roots %
*Agathophora alopecuroides*	-	5.6	5.6	16.6
*Anabasis setifera*	-	8.4	14	25.1
*Atriplex halimus* L	-	-	-	-
*Halocnemum strobitaceum*	-	5.6	5.6	-
*Lantana camara* L	5.6	-	5.6	-
*Mesembryanthemum forsskaollii*	19.5	5.6	8.4	-
*Raphanus raphanistrum*	-	8.4	2.8	16.8
*Suaeda vermiculata*	-	-	-	25.0
*Zygophyllum coccineum*	-	5.6	2.8	-

### Colonization frequency rate and diversity of EF

The evaluation of fungal occurrence in isolated plant tissues was calculated by colonization frequency rate (CF %). Moreover, the colonization rate in different plant tissues ranged from 2.8 to 25.1%. The higher colonization rate was reported for *A. setifera* root tissues (25.1%) and *S. vermiculata* root (25%), which were close, followed by *M. forsskaollii* flowers (19.5%), and *R. raphanistrum* root were (16.8%). The lowest value appeared (2.8%) in both *Z. coccineum* and *R. raphanistrum* stems, as shown in Table 2. *A. setifera* (a xerophytic desert shrub) is classified as a chamaephyte. However, its typical habitat in the PAWD is primarily the midstream zone, with occasional extension into the downstream under specific conditions^26,43^. Aletaha et al.^44^ observed that the frequency of root EF can be higher in dried soils with pH values (7–8) and high altitudes than in temperate soils.

**Fig. 3:**
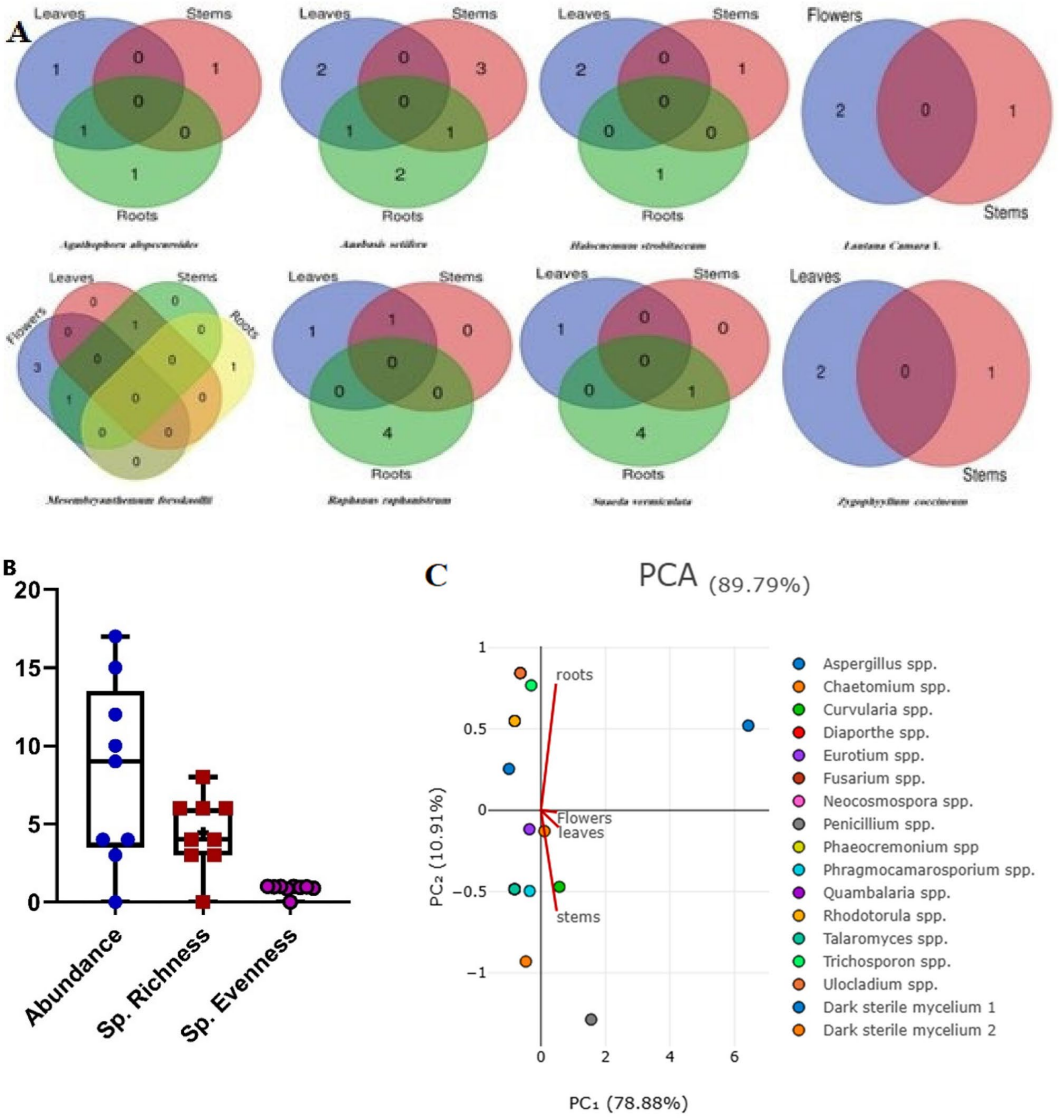
Distribution and diversity of EF in different selected plant tissues (**A**) Venn diagram showing overlaps of EF recovered from flowers, leaves, stems, and roots. The numbers correspond to the number of species of EF in plant tissues, (**B**) Boxplots displaying abundance, species richness, and species evenness of EF isolated from selected plants, (**C**) Principal component analysis (PCA) showing the distribution of FE genera isolated according to different tissues of different plants.

The species diversity of EF was measured by the diversity indices, which are species richness and relative species abundance. To characterize the diversity of the isolated EF, we calculated several biodiversity indices, Simpson’s (D`), Shannon-Wiener (H`), Camargo’s (1/S), and Berger-Parker (BP) to confirm results as shown in Table 3. Similar trends observed in the Shannon-Wiener and Simpson’s diversity indices, the values were the highest in *A. setifera* (H`= 1.79, 1-D`=0.789), and that of *S. vermiculata* and *R. raphanistrum* were the same (H`= 1.74, 1-D`=0. 0.82), followed by *M. forsskaollii* (H`= 1.59, 1-D`=0. 0.75). Similarly, Aletaha et al.^44^ showed a highly significant Shannon’s diversity index found in *A. setifera*. The Camargo’s index revealed that the species diversity in *S. vermiculata*,* M. forsskaollii*, and *R. raphanistrum* (1/S = 0.16) was higher than in *A. setifera* (1/S = 0.125). While the lowest index (Simpson’s, Shannon’s diversity, and Camargo’s indices) appeared in *L. camara* (H`= 1.041, 1-D`= 0.625, 1/S = 0.33) and *Z. coccineum* (H`= 1.1, 1-D = 0.667, 1/S = 0.33). The Berger-Parker dominance index was the highest for *L. camara*. Since one species accounts for the majority of its community, this indicates significant dominance^45^. Figure 3 A illustrated the richness of EF isolated from various plant tissues, showing that *A. setifera* gave the largest numbers of EF among its different tissues. Moreover, *A. setifera* demonstrated the highest abundance (17) and the highest species richness (9.0) with species evenness (0.861) as highlighted in Fig. 3B. Principal component analysis (PCA) indicated the distribution of FE genera in different plant tissues as shown in Fig. 3 C. Our results agreed with Park et al.^46^ revealed that Shannon diversity index and richness in the different root tissues are higher than stem and leaf tissues, and also certain fungal endophytes are specific to tissue. Chen et al.^13^ showed that the diversity and richness of the FE community was significantly influenced by different locations and seasons Environmental variation may influence fungal diversity, such as temperature, relative humidity, rainfall, location and growth of plant stage^47,48^. According to our knowledge, as far as the authors are aware, the present investigation might be the first study on the diversity of EF from these medicinal plants in Egypt. More future prospects will done to elucidate the EF diversity using metagenomics.

**Table 3:** Diversity indices of EF from selected medicinal plants.

Host plants	Shannon diversity H”	Simpson’s diversity index (1-D`)	Camargo’s index (1/S)	Berger parker index (BP)
*Agathophora alopecuroides*	1.46	0.738	0.25	0.4
*Anabasis setifera*	1.79	0.789	0.125	0.35
*Atriplex halimus* L	-	-	-	-
*Halocnemum strobitaceum*	1.39	0.75	0.25	0.25
*Lantana camara L*	1.041	0.625	0.33	0.5
*Mesembryanthemum forsskaollii*	1.59	0.75	0.16	0.41
*Raphanus raphanistrum*	1.74	0.82	0.16	0.2
*Suaeda vermiculata*	1.74	0.82	0.16	0.22
*Zygophyllum coccineum*	1.1	0.667	0.33	0.33

### Antimicrobial activity

Ethyl acetate extracts (EAEs) of 33 different EF isolated from selected medicinal plants were screened for their antimicrobial activities at a concentration of 150 µg/ml by an agar well diffusion assay against nine pathogenic (MDR) strains: *S. aureus*, *P. aeruginosa*,* K. pneumonia*,* E*. *coli*, *C. albicans*,* C. kefyr*,* C. krusei*,* Penicillium* sp., and *R. stolonifer*.

**Fig. 4:**
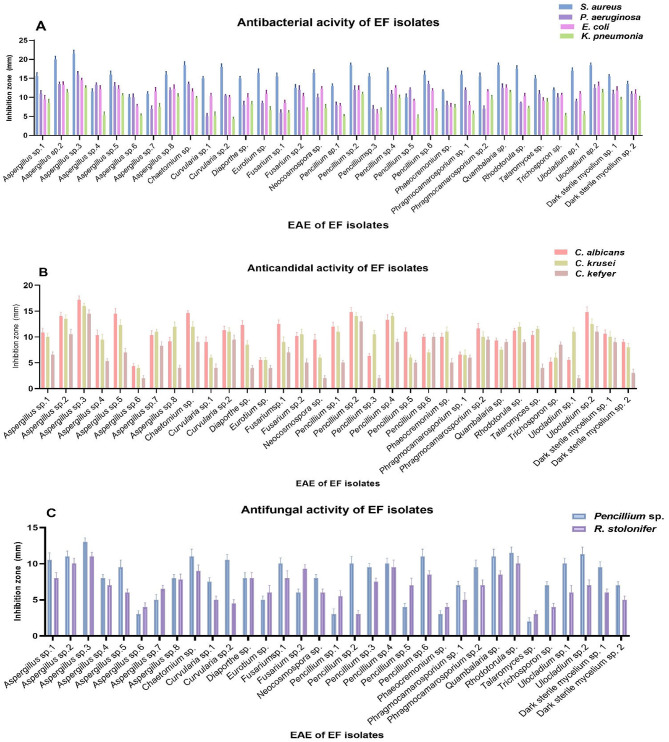
**(A)** Antibacterial activity of EAEs of EF isolated from selected plants against pathogenic bacteria strains, **(B)** Anticandidal activity of EAEs of EF isolated from selected plants against pathogenic *Candida* strains, **(C)** Antifungal activity of EAEs of EF isolated from selected plants against pathogenic fungi strains.

Figure 4(A) showed the antibacterial activity of EAE from FE against pathogenic bacterial strains. Among these, *Aspergillus* sp.3 exhibited the largest inhibition zone, indicating the strongest antibacterial efficacy, followed by *Aspergillus* sp.2 (isolated from *A*. *setifera*), *Penicillium* sp.2 (also from *A. setifera*), *Ulocladium* sp.2 (from *S. vermiculata*), and *Chaetomium* sp. (associated with *A. alopecuroides*). Similarly, Fig. 4(B) highlighted the anti-candidal potential of these extracts, with *Aspergillus* sp.3 showing the highest activity against pathogenic Candida strains, followed by *Penicillium* sp.2 (from *A. setifera*), *Ulocladium* sp.2 (from *S. vermiculata*), *Aspergillus* sp.2 (also from *A. setifera*), and *Aspergillus* sp.5 (isolated from *H. strobilaceum*). Finally, Fig. 4(C) revealed the antifungal activity of the EAE, where *Aspergillus* sp.3 again displayed the largest inhibition zone. Subsequent efficacy was observed for *Rhodotorula* sp. (associated with *R. raphanistrum*), *Aspergillus* sp.2 (from *A. setifera*), *Chaetomium* sp. (from *A. alopecuroides*), and *Penicillium* sp.6 (isolated from *S. vermiculata*). Our results were in agreement with those obtained by Hashem et al.^49^ who revealed that *Aspergillus* sp. endophyte had 16 major bioactive metabolites with a wide range of pharmacological properties, which demonstrated antifungal effect against fungi causing mucormycosis. Furthermore, Kaur and Arora^50^ isolated endophytic *Chaetomium* sp. from *Moringa oleifera* displaying a broad-spectrum antimicrobial efficiency against the used strains.

### Antioxidant activity

**Fig. 5:**
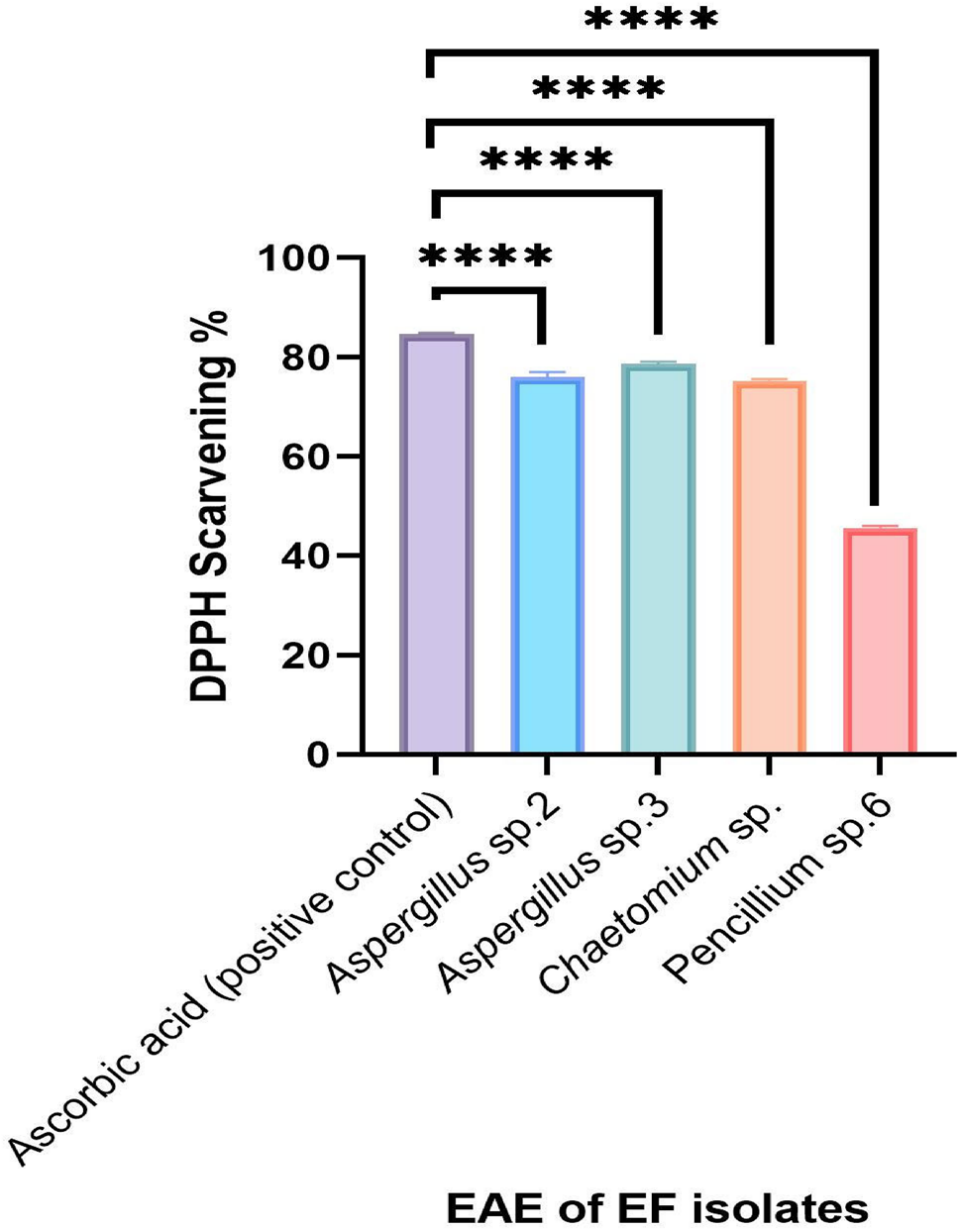
Antioxidant activity of EAE of EFs compared with ascorbic acid.

The antioxidant activity of EAEs was evaluated by 2,2-diphenyl-1-picrylhydrazyl (DPPH) free radical scavenging activity. From our result, only 4 species out of 33 isolated endophytes showed antioxidant activity. Figure 5 revealed that EAE of *Aspergillus* sp. 3 (78.17%) exhibited significantly the highest antioxidant activity, followed by *Aspergillus* sp. 2 extract (76.8%), *Chaetomium* sp. extract (75.42%), and *Penicillium* sp. 6 extract (45.1%) as compared to standard ascorbic acid. Our results were in agreement with those obtained by Saleh et al.^51^ who reported the antioxidant activity of *Aspergillus* sp. endophyte. Additionally, Khalil et al.^52^ showed the antioxidant activity of *Chaetomium* sp. endophyte. Furthermore, Abdel Razek et al.^22^ isolated endophytic *Penicillium* sp. associated with *H. strobilaceum* that had antioxidant, antimicrobial, and antibiofilm activities.

### Molecular identification of the most potent EF

**Fig. 6:**
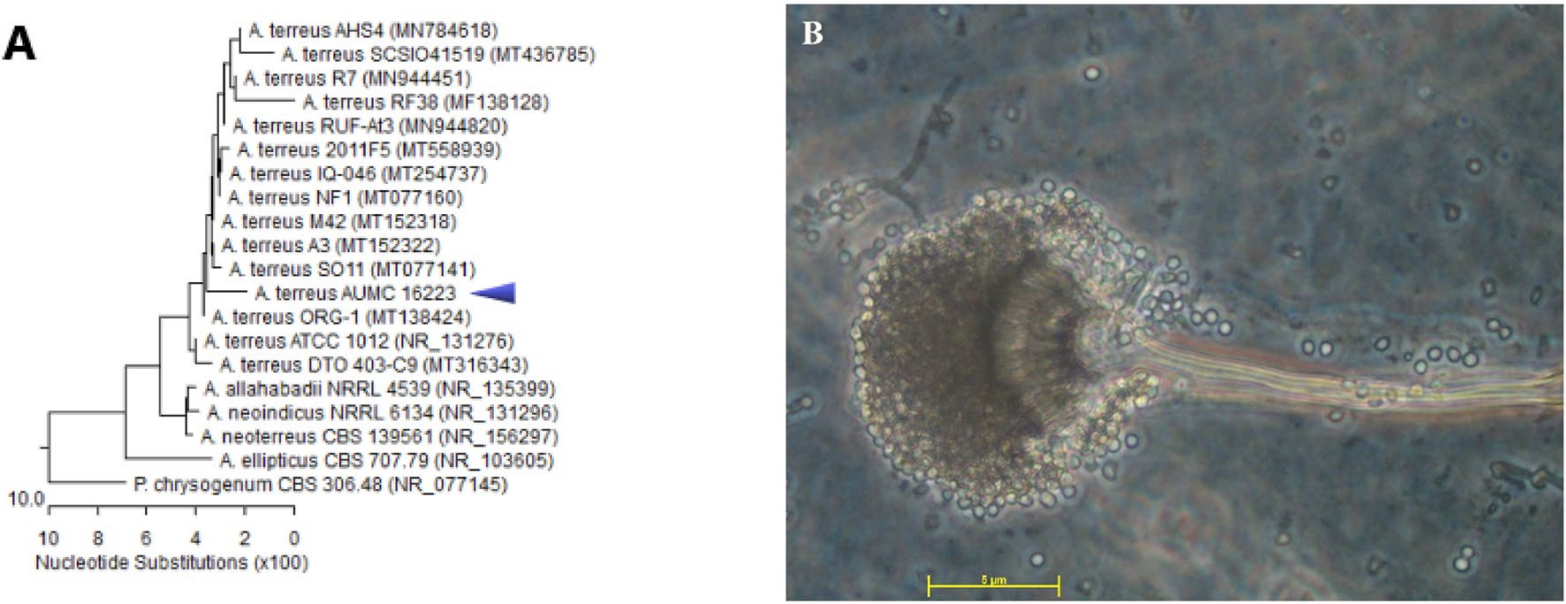
(**A)** Phylogenetic tree of *A. terreus* AUMC16223 with accession number PP491988 (arrowed). (**B**) Examination of *A. terreus* AUMC16223 under the upright light microscope (Zeiss, AXIO Imager. M1) with 1000x magnification.

The most potent antioxidant and antimicrobial isolate was reidentified by molecular sequencing of the ITS region with universal primers ITS1 and ITS4 as *Aspergillus terreus* AUMC16223 with accession number PP491988 with closely related strains accessed from the GenBank (Fig. 6). Sequencing of the ITS region revealed that this isolate showed 98% identity and 100% coverage with several strains of the same species, including the type material *A. terreus* ATCC 1012 with accession no. NR131276.

### Cytotoxicity assay

The first step to ascertain the safety of bioactive metabolites on noncancerous human cells is to evaluate their cytotoxicity^53^. The most potent EF *Aspergillus terreus* AUMC16223 was then assessed for cytotoxic activity against cell lines of normal human lung fibroblasts (WI38) and cancerous human lung cells (A549) by MTT assay. The treatment of cell lines with EAEs of EF at several concentrations (1–500 µg/mL) resulted in a decline in the cancerous cell viability significantly, while EAEs of EF showed a nontoxic effect against the normal cell line^54^.

**Fig. 7:**
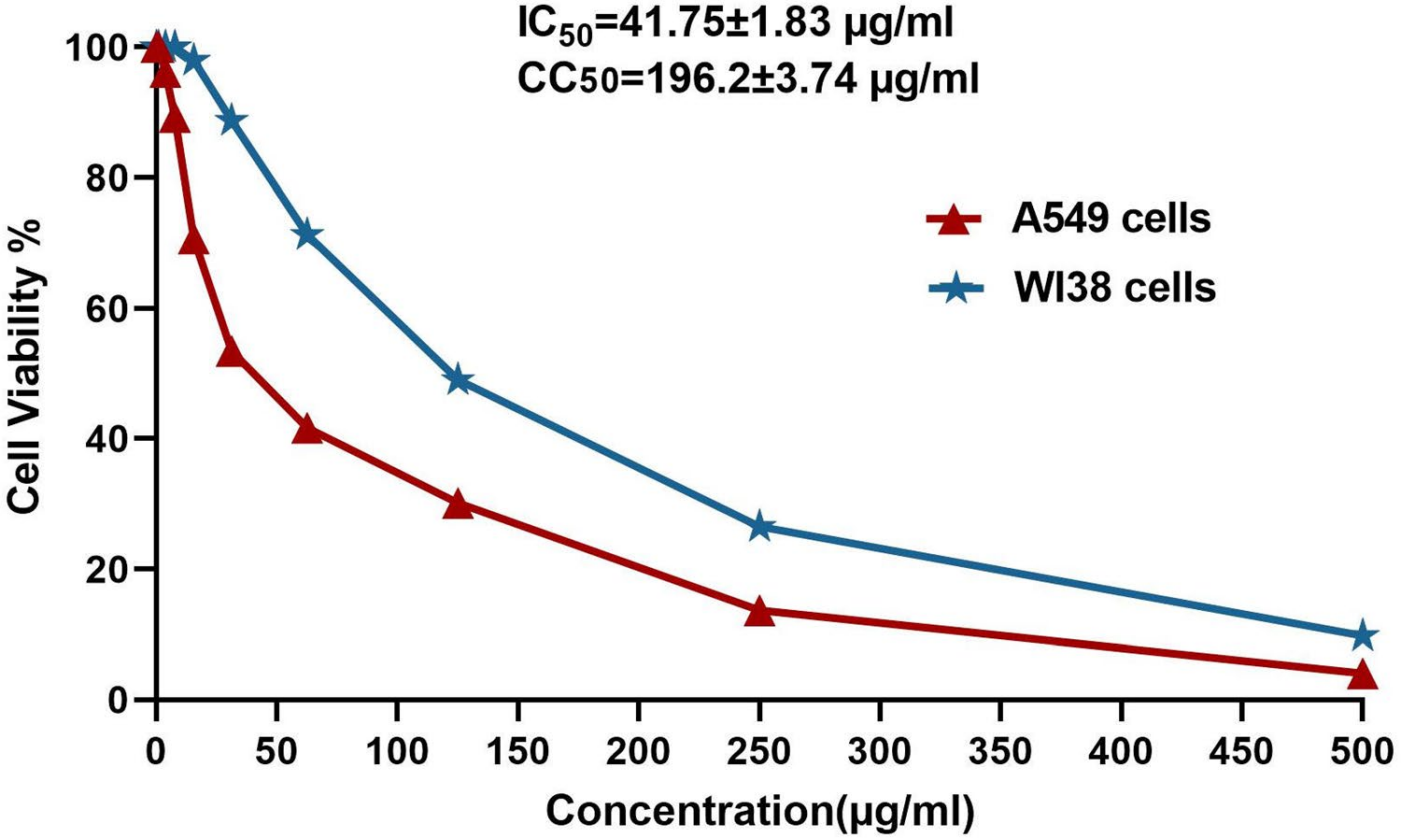
Cell viability analysis of A549 cells (Human lung cancer cell line) and WI38 cells (human lung fibroblast normal cell line).

The ethyl acetate extract of *A*. *terreus* endophyte had a very strong impact on the cancer cell lines that were tested, with a significant SI value of 4.69 indicating that they had a high antitumor potential^55^. Overall, the current study revealed that the extract of the *Aspergillus terreus* endophyte showed anticancer potential activity against cancer cells (A549) with an IC_50_ value of 41.75 ± 1.83 µg/ml and nontoxic effect on the normal cell line (WI 38) with a significant CC_50_ value 196.2 ± 3.74 µg/mL, as shown in Fig. 7. Similarly, Rustamova et al.^56^ revealed that endophytic *A. terreus* showed its cytotoxicity against MDA-MB 231 (breast cancer), HT29 (colon cancer), and Hela (cervical cancer) cell lines. Moreover, Gupta et al.^25^ isolated bioactive compound terrain from *A. terreus* endophyte showing cytotoxicity, antimicrobial, and biocontrol agents for inhibition of certain phytopathogenic fungi. Furthermore, Ukwatta et al.^57^ isolated the bioactive metabolite cowabenzophenone A from *A. terreus* endophyte showing antimicrobial, anti-filarial, alpha-glucosidase inhibitory, anti-inflammatory, and cytotoxic activities.

Although there are several reports on endophytic *A. terreus*^25,36,57–60^, our study is the first one to isolate *A. terreus* associated with *Anabasis setifera*,* Mesembryanthemum forsskaollii*,* Raphanus raphanistrum*, and *Zygophyllum coccineum* in Egypt and study its biopotential activities.

### Conclusion

This study described the diversity of EF found in selected medicinal plants and emphasized their biopotential activities, showing *Aspergillus terreus* endophyte extract as the most significant antimicrobial, antioxidant, and cytotoxic agent. More research on endophytic *A. terreus* associated with these selected medicinal plants for bioactive chemical components and characterization will be further investigated to find alternative drugs as a promising source of therapeutic agents.

## Methods

### Plant samples collection

**Fig. 8:**
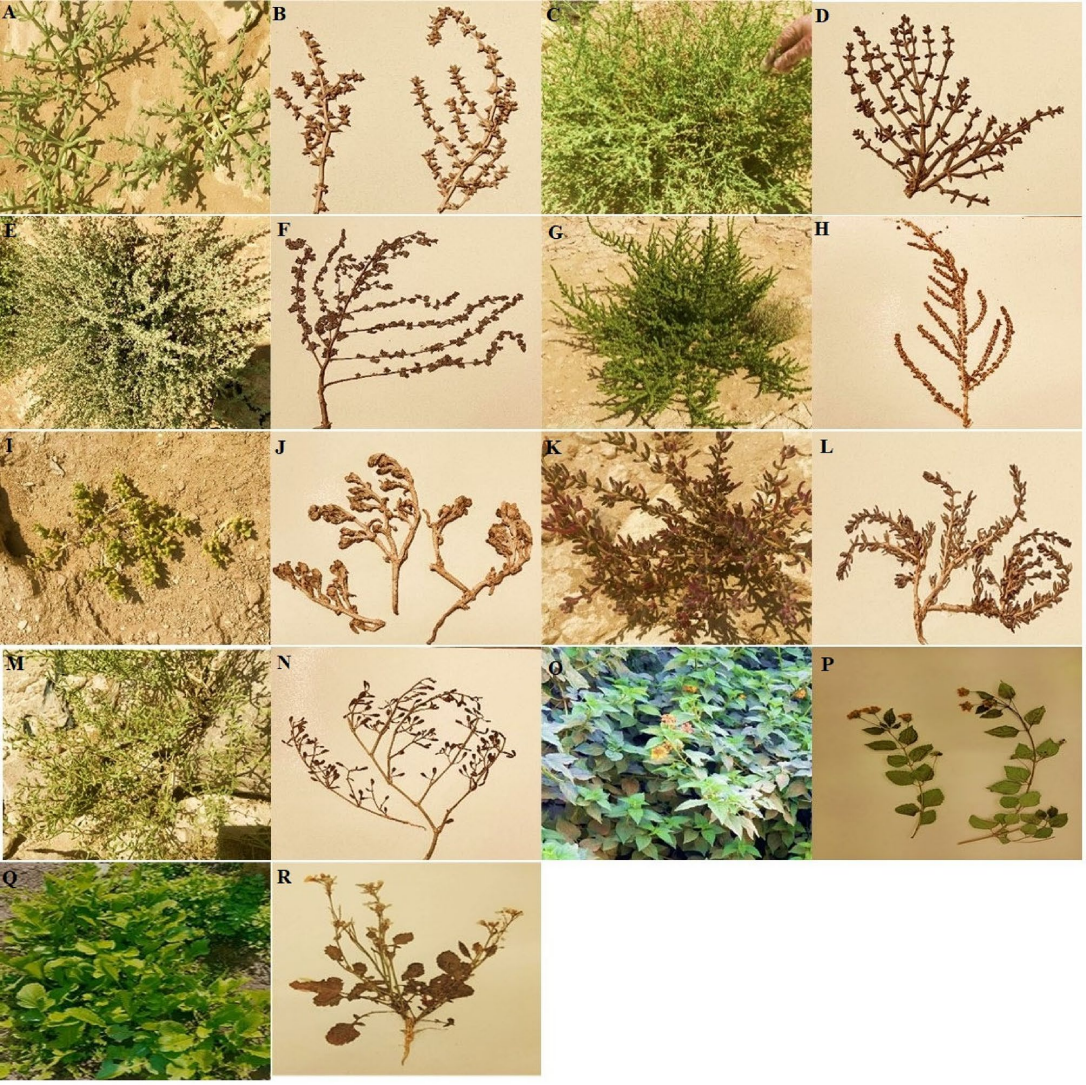
Selected medicinal plants (**A**) *Agathophora alopecuroides*, (**B**) Preserved *A. alopecuroides*, (**C**) *Anabasis setifera*,* (***D***)* Preserved *A. setifera*, (**E***) Atriplex halimus* L, (**F**) Preserved *A. halimus* L, (**G**) *Halocnemum strobitaceum*,* (***H***)* Preserved *Halocnemum strobitaceum*, (**I**) *Mesembryanthemum forsskaollii*, **(J)** Preserved *M. forsskaollii*,* (***K***) Suaeda vermiculata*, (**L**) Preserved *S. vermiculata*, (**M**) *Zygophyllum coccineum*, (**N**) Preserved *Z. coccineum*,* (***O***) Lantana camara* L., (**P**) Preserved *L. camara* L., (**Q**) *Raphanus raphanistrum subsp. sativus.*, **(R)** Preserved *R. raphanistrum subsp. sativus.*

Nine cultivated and wild plant medicinal samples were collected in March and June 2021 from the Natural Cultivated Area in Helwan (NCAH) and the Protected Area of Wadi Degla in Maadi, Egypt (PAWD), respectively. These plants were *Agathophora alopecuroides*,* Anabasis setifera*,* Atriplex halimus* L, *Halocnemum strobitaceum*,* Mesembryanthemum forsskaollii*, *Suaeda vermiculata*, *Zygophyllum coccineum*,* Lantana camara*, and *Raphanus raphanistrum subsp. sativus* as in Fig. 8. Fresh, healthy plant samples were placed in a plastic sealed bag and kept at 4 °C until the isolation procedure occurred within 48 h of collection, and samples were identified based on botanical characteristics by Prof. Dr. Loutfy Mohsen Hassan, Department of Botany, Faculty of Science, Helwan University^27,61,62^. A voucher’s plant specimens were kept at the Helwan University Herbarium (HUH), Faculty of Science in Egypt, as shown in Table 1.

### Isolation of EF

**Fig. 9:**
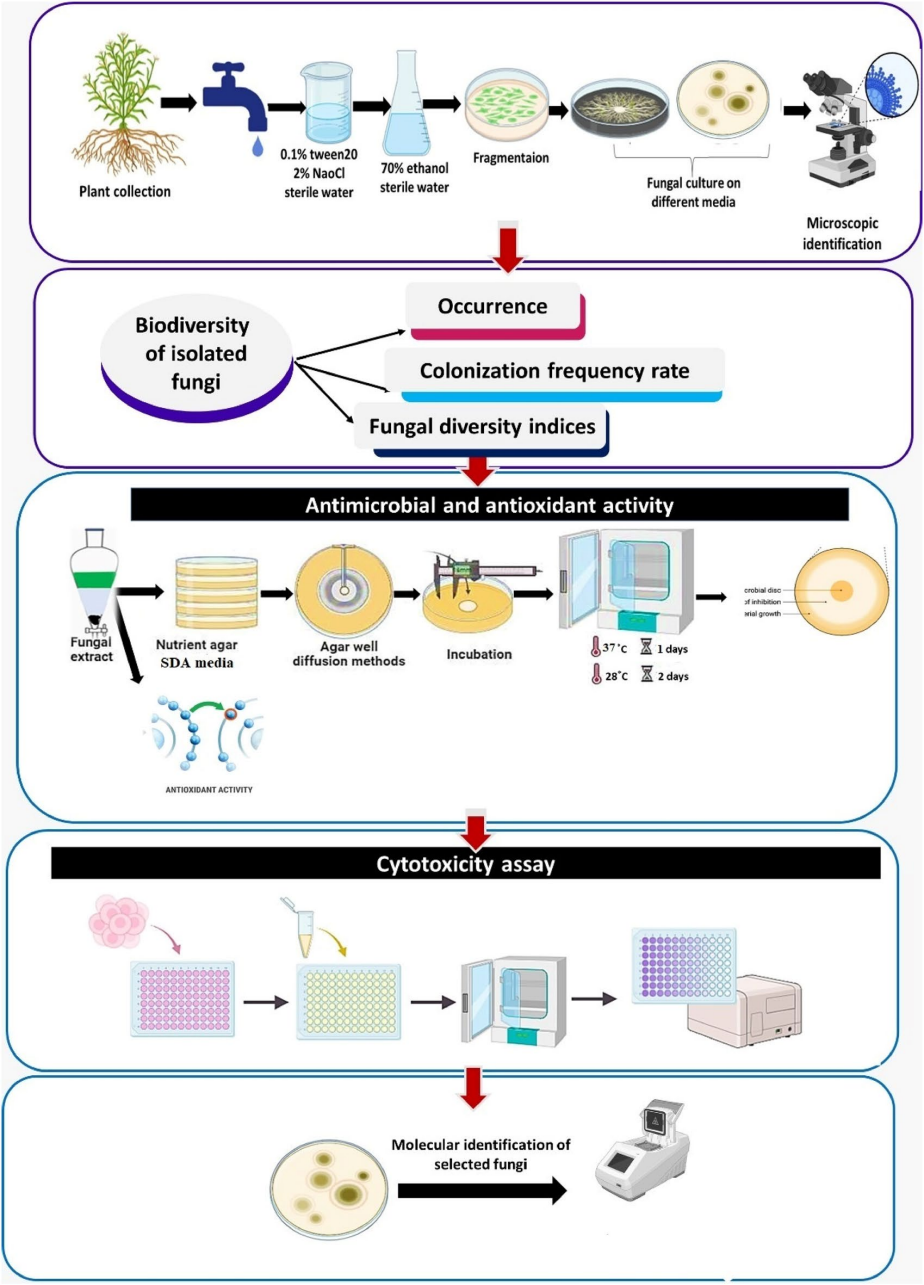
Experimental design used in this study.

Isolation of EF required surface sterilization of plants to remove dust and get rid of epiphyte^63,64^. Healthy plants were separated into four tissues (flowers, leaves, stems, and roots), washed, and cut into small fragments as in Fig. 9. Surface sterilization involved immersion in 0.1% Tween 20 for 30 s, 1–3 min in 2% sodium hypochlorite, and rinsing with sterilized distilled water. This was followed by two minutes in 70% ethanol and another rinse. These sterilized tissue samples were dried on sterilized filter papers in a laminar airflow chamber. Subsequently, cultured on different agar plates of enriched and semi-selective media, potato dextrose agar (PDA), sabouraud dextrose agar (SDA), and dichloran glycerol (DG18) agar (Merck–Germany). Antibacterial chloramphenicol (100 mg/l) was added. One milliliter of the final rinse water was cultured on agar plates (control) to verify the qualification of the surface tissue sterilization method. Media plates with sterilized tissue samples were incubated for 7–14 days at 28ºC. Colony purification was achieved by subculturing the colonies on various agar plates until pure isolates were obtained. For further use, the fungal isolates were kept on a PDA slant at 4ºC.

### Morphological identification of EF

Both macroscopic and microscopic features of all EF isolates were recognized primarily using the identification standards manual^65–69^ at Assiut University Mycological Center (AUMC), Egypt. A variety of culture media (PDA-Czapeck’s agar-Malt extract agar) were used to demonstrate the fungal colonies morphology (e.g., color, mycelia, hyphae, margin, texture, growth rate, etc.) for macroscopic identification. The staining method with lactophenol cotton blue was applied to slides from the cultures to visualize hyphae, conidiophores, conidia, their arrangement, and spore characteristics for the identification of microscopic features. The identification of yeast was carried out by the API *Candida* test (bioMérieux Vitek, Hazelwood, MO, USA).

### Evaluation of EF occurrence and diversity

The percentage of occurrence of each EF isolate was calculated according to the method described by Heino^70^.

$$\:\text{Occurrence} \:\text{of}\:\text{EF}\:=\frac{\text{Number}\:\text{of}\:\text{colonies}\:\text{of}\:\text{species}}{\text{Total}\:\text{number}\:\text{of}\:\text{isolated}\:\text{colonies}}\times\:100$$

Colonization frequency percentage (CF%) was utilized to compare the degrees of EF isolates between different plant tissues, calculated according to the method described by Suryanarayanan et al.^71^$\:\text{C}\text{F}{\%}=\frac{\text{N}\text{o}.\:\text{o}\text{f}\:\text{s}\text{e}\text{g}\text{m}\text{e}\text{n}\text{t}\text{s}\:\text{c}\text{o}\text{l}\text{o}\text{n}\text{i}\text{z}\text{e}\text{d}\:\text{b}\text{y}\:\text{E}\text{F}}{\text{T}\text{o}\text{t}\text{a}\text{l}\:\text{N}\text{o}.\:\text{o}\text{f}\:\:\text{p}\text{l}\text{a}\text{n}\text{t}\:\text{s}\text{e}\text{g}\text{m}\text{e}\text{n}\text{t}\text{s}}\times\:100$

The differences in EF communities identified at different tissues of each plant were analyzed using Venn diagrams and Principal component analysis (PCA).

Species diversity was incorporated into both species number in a certain plant and the evenness of their abundance. Where abundance (N) was calculated as the total species number in one plant. Species richness (S) was the number of different species in one plant, and the relative abundance (evenness (E)) was calculated as$$\:E=\raisebox{1ex}{$\text{H}`$}\!\left/\:\!\raisebox{-1ex}{$\text{ln}\text{s}$}\right.$$ where H` is the Shannon index and S is the species number in the community (richness).

The diversity of EF was calculated by multiple diversity indices: Shannon–Wiener H, Simpson’s D, Camargo’s, and Berger–Parker BP for each plant.

Camargo’s index$$\:=\raisebox{1ex}{$1$}\!\left/\:\!\raisebox{-1ex}{$S$}\right.$$ was calculated according to Camargo^72^.

Shannon Diversity (H`) was calculated according to Shannon & Weaver^73^.

$\:H`=-{\sum\:}_{k\:=\:0}^{n}{p}_{i}\text{ln}{p}_{i}$ Where $\:{p}_{i}$ is the percent of the number of a certain EF strain to the total number.

Also, Simpson’s Diversity Index (D`) was calculated according to Simpson^74^.

$\:D`=1-\sum{\left(\frac{n}{N}\right)}^{2}$ Where N is the total number of all species, n is the total number of one species.

Moreover, the Berger–Parker index BP was calculated according to Berger and Parker^75^.

$\:\text{B}\text{P}=\frac{\text{N}\text{m}\text{a}\text{x}}{\text{N}}$ Where N is the total number of species, N_max_ is the most abundant species.

### Fermentation and extraction of secondary metabolites

Three mycelial discs of each EF strain were inoculated into 100 mL potato dextrose broth (PDB) media in a 250 mL Erlenmeyer flask. Flasks were incubated under static conditions at 28 ± 2^°^C for fourteen days. After incubation, filtration of fungal cultures was performed to remove mycelia. After that, ethyl acetate extraction of the filtrate was performed three times by the separating funnel with vigorous shaking and then collecting the upper organic layer^23^. After evaporation, ethyl acetate extracts (EAEs) (150 µg/ml) were kept at 4 °C for further bioactivity screening.

### Microbial strains

Assessment of the antimicrobial activity of EAEs of EF was performed against nine pathogenic MDR strains of bacteria, yeasts, and fungi: *E*scherichia *coli*, *Klebsiella pneumonia*,* Pseudomonas aeruginosa*,* Staphylococcus aureus*, *Candida albicans*,* Candida kefyr*,* Candida krusei*, *Penicillium* sp., and *Rhizopus stolonifer* collected from our previous studies^76–81^. Each strain was subcultured, and the concentration was adjusted to (1 × 10^5^ CFU/ml) with sterile saline solution.

### Biomedical assessments

#### Antimicrobial activity

Each EF extract was assessed for its antimicrobial activity against selected pathogenic MDR strains. This assay was performed by the agar well diffusion method^82^. Nutrient agar (NA Merck) medium was used for bacteria, and SDA was used for yeasts and fungi. By using the pour plate technique, each strain suspension (1 × 10^5^ CFU/ml) was mixed with melted agar media. The cork borer was used to create an appropriate well on a solidified agar plate. In each well, EAEs (150 µg/ml) were added separately and left to stand for 10 min. Then plates were incubated for 24 h for bacteria at 37ºC and 24–72 h for yeast and fungi at 28ºC. The inhibition zone diameter of three triplicates was measured in millimeters and compared to the control (solvent only) to determine antimicrobial activities.

#### Antioxidant activity

Each EF extract was measured for the free radical scavenging potential as described by Wang et al.^83^ modified by El Barky & Mohamed^84^. Twenty-five microliters of each EAE were added individually to 975 µL methanolic DPPH. The mixtures were homogenized and kept at room temperature in the dark. The reference standard was ascorbic acid (50 µg mL^−1^). A UV-90 spectrophotometer (Taisite - USA) was used to measure the mixture at 517 nm. The percentage of DPPH scavenging activity of each sample was calculated using the formula.$\:=\frac{\text{C}-\text{T}}{\text{C}}\times100$ Where T is the sample absorbance and C is the control absorbance.

### Molecular identification and examination of the most potent EF

Molecular techniques were used to confirm the identification of the most potent EF isolate by sequencing the internal transcribed spacer (ITS) of ribosomal DNA at the Assiut University Mycological Center (AUMC), Egypt. The sequencing of ribosomal RNA genes (rDNA) was performed by SolGent Company (Daejeon, South Korea). The PCR amplification of ITS was done by universal primers: ITS1 (5’-TCC GTA GGT GAA CCT GCG G-3’) and ITS4 (5’-TCC TCC GCT TAT TGA TAT GC-3’). The resulting sequences were analyzed using the Basic Local Alignment Search Tool (BLAST) from the National Center of Biotechnology Information (NCBI) website to find the homology with the closest related organisms (http://www.ncbi.nlm.nih.gov). It was subjected to the Clustal W analysis by MegAlign software version 5.05 (DNASTAR Inc., Madison, Wisconsin, USA) for the phylogenetic analysis. Also, this isolate was examined under an upright light microscope (Zeiss, AXIO Imager.M1, Germany) at the Grand Egyptian Museum-Conservation Center (GEM-CC), Giza, Egypt.

### Cytotoxicity activity

Evaluation of EAE of the most potent EF against non-tumor and tumor cell lines, namely a normal human lung fibroblast cell line (WI 38) and a carcinoma human lung cell line (A549) at the Fermentation Biotechnology & Applied Microbiology Center, Azhar University, Egypt.

The culture media for cell A549 was RPMI-1640 with 50 µg/ml gentamycin and 10% inactivated fetal calf serum, and that of cell WI38 was Dulbecco’s modified Eagle’s medium (DMEM) with 1% L-glutamine, HEPES buffer, 50 µg/ml gentamycin, and 10% heat-inactivated fetal bovine serum. Two times a week, all cells were subcultured at 37ºC in a humidified atmosphere with 5% CO_2_. Briefly, each cell was seeded in microtiter plates (96 wells, 100 µL/well) and incubated for 24 h. Different EAE concentrations of 1–500 µg/mL of selected EF were tested. Cells were treated with each experimental group (100 µL/well) to determine their cytotoxicity in comparison to untreated cells. The cell viability and cytotoxicity percentage were calculated according to the following Eq^57^.:$\:\:\:\text{V}\text{i}\text{a}\text{b}\text{i}\text{l}\text{i}\text{t}\text{y}{\%}=\frac{\text{a}\text{b}\text{s}\text{o}\text{r}\text{b}\text{a}\text{n}\text{c}\text{e}\:\text{o}\text{f}\:\text{t}\text{h}\text{e}\:\text{t}\text{r}\text{e}\text{a}\text{t}\text{e}\text{d}\:\text{s}\text{a}\text{m}\text{p}\text{l}\text{e}}{\text{a}\text{b}\text{s}\text{o}\text{r}\text{b}\text{a}\text{n}\text{c}\text{e}\:\text{o}\text{f}\:\text{u}\text{n}\text{t}\text{r}\text{e}\text{a}\text{t}\text{e}\text{d}}\times100$

$$\:\text{C}\text{y}\text{t}\text{o}\text{t}\text{o}\text{x}\text{i}\text{c}\text{i}\text{t}\text{y}\:{\%}=100-\:\text{V}\text{i}\text{a}\text{b}\text{i}\text{l}\text{i}\text{t}\text{y}{\%}$$

A dose-response curve for each concentration was plotted in GraphPad Prism software (San Diego, CA, USA) to determine the IC_50_ value (concentration needed to inhibit 50% of cell growth), while the concentration needed to cause toxic effects in 50% of normal cells was CC_50_.

Selectivity Index (SI) was determined for each treatment; it was divided by CC_50_ normal cells by that of IC_50_ of tumor cells. It was considered that a value above 3 reported high selectivity^85^. SI was calculated by the following formula^55^$\:\text{S}\text{I}=\:\frac{\text{C}\text{C}50\:\left(\text{W}\text{I}38\:\text{c}\text{e}\text{l}\text{l}\text{s}\right)}{\text{I}\text{C}50\left(\text{A}549\:\text{c}\text{e}\text{l}\text{l}\text{s}\right)}.$

### Statistical analysis

Analysis of variance (ANOVA) one-way tests by SPSS, version 20 (IBM, Armonk, USA) to estimate the statistical parameters. Multiple range tests of Duncan’s were performed at a significance level of *P* = 0.05^86^. GraphPad Prism software 9.3.1 (GraphPad Software, Inc., CA, USA) was used for graphical representations.

## Data Availability

Data availabilityThe datasets of RNA sequences analyzed during the current study are available at the Assiut University Mycological Center (AUMC), Egypt with accession number PP491988.

## Abbreviations

EF: Endophytic Fungi
EAE: Ethyl Acetate Extract
CF: Colonization Frequency
NCAH: Natural Cultivated Area in Helwan
PAWD: Protected Area of Wadi Degla
